# Assessing the role of volumetric‐modulated arc therapy in hepatocellular carcinoma

**DOI:** 10.1120/jacmp.v14i3.4162

**Published:** 2013-05-06

**Authors:** Mian Xi, Li Zhang, Qiao‐Qiao Li, Lei Zhao, Rui Zhang, Meng‐Zhong Liu

**Affiliations:** ^1^ State Key Laboratory of Oncology in Southern China Sun Yat‐sen University Guangzhou China; ^2^ Department of Radiation Oncology, Cancer Center Sun Yat‐sen University Guangzhou China; ^3^ Department of Radiation Oncology The First Affiliated Hospital of Kunming Medical University Kunming China

**Keywords:** hepatocellular carcinoma, volumetric‐modulated arc therapy, three‐dimensional conformal radiotherapy, dosimetry

## Abstract

The role of volumetric‐modulated arc therapy (VMAT) in hepatocellular carcinoma (HCC) remains controversial. The purpose of this study was to determine the potential clinical role of VMAT compared with three‐dimensional conformal radiotherapy (3D CRT) for liver irradiation. Four‐dimensional CT scans of 24 patients with unresectable HCC were included and divided into two groups: (1) adjacent group (n=11), with planning target volumes overlapping or within 1 cm adjacent to the alimentary tract; (2) nonadjacent group (n=13), in which the normal liver itself was the dose‐limiting structure. Target coverage, organs‐at‐risk (OARs) doses, delivery parameters, and treatment accuracy were evaluated. Superior target coverage, conformity, and homogeneity were achieved with VMAT compared with 3D CRT. In the adjacent group, VMAT provided superior sparing of serial functioning OARs including the stomach, small intestine, and spinal cord. In the nonadjacent group, VMAT provided inferior sparing of most OARs including the liver, stomach, and small intestine. For the whole group, the effective treatment time was 2.1±0.3 min for 3D CRT and 3.1±0.2 min for VMAT. For liver lesions adjacent to the alimentary tract, this study indicates that VMAT should be selected due to the plan quality, delivery efficiency, and superior sparing of stomach and small intestine. However, for liver lesions away from the alimentary tract, VMAT is not superior to 3D CRT for normal tissue protection.

PACS number: 87.55‐x.

## I. INTRODUCTION

With advances in radiotherapy modalities, radiation therapy has gained increasing importance in the management of unresectable hepatocellular carcinoma (HCC).^1^, ^2^ Various techniques are available for liver irradiation, such as three‐dimensional conformal radiotherapy (3D CRT), intensity‐modulated radiotherapy (IMRT), and volumetric‐modulated arc therapy (VMAT).^3^, ^4^, ^5^ Previous studies have shown that, compared with 3D CRT, IMRT can reduce the dose to critical organs in the upper abdomen while maintaining target coverage for pancreatic and gastric cancers.^6^, ^7^ Our recent study showed that IMRT and VMAT were superior to 3D CRT in target coverage and sparing of most organs at risk (OARs) in patients with abdominal lymph node metastasis from HCC.^(8)^ However, the role of IMRT/VMAT in HCC itself remains controversial. Cheng et al.^(9)^ reported that IMRT achieved acceptable target coverage for HCC, but had a negative dosimetric effect on the liver, with a significant increase in mean dose compared with 3D CRT. Nevertheless, Eccles et al.^(10)^ suggested that IMRT improved planning target volume (PTV) coverage while maintaining normal tissue tolerance in most 3D CRT liver plans. Therefore, there is no accepted standard strategy for liver irradiation.

The major challenge of radiotherapy for HCC is the presence of multiple critical structures, including the normal liver, stomach, small intestine, kidneys, and spinal cord. Dose‐limiting factors can differ according to the location of the tumor within the liver. Because the optimal irradiation technique is likely to depend on the location of the liver lesion, it is important to investigate cases in which IMRT or VMAT might offer benefit compared with 3D CRT. Because VMAT is known to provide superior target coverage and similar sparing of OARs in HCC compared with IMRT,^5^, ^8^, ^11^ we selected VMAT rather than IMRT to compare with 3D CRT in this study.

The purpose of the study was to evaluate the potential clinical role of VMAT compared with 3D CRT, and to identify the optimal irradiation technique for different groups of unresectable HCC patients.

## II. MATERIALS AND METHODS

### A. Patient inclusion criteria and characteristics

Four‐dimensional computed tomography (4D CT) scans of 24 locally advanced HCC patients who were previously treated with 3D CRT between June, 2008 and December, 2011 were selected for this retrospective comparative analysis. The inclusion criteria were: (1) age 20–70 years; (2) histopathologically proven HCC, surgically unresectable or unsuitable for resection; (3) Child–Pugh A liver function and disease confined to one lobe of the liver; (4) more than 800 cc of uninvolved liver; (5) Karnofsky performance score ≥70; and (6) tumor mobility in the craniocaudal direction less than 1.5 cm.

Patient and tumor characteristics are listed in Table 1. The patients were divided into two groups: (1) an adjacent group (n=11), in which PTVs overlapped or were within 1 cm adjacent to the alimentary tract (stomach and small intestine); and (2) a nonadjacent group (n=13), in which the normal liver itself was the critical dose‐limiting structure.

**Table 1:** Patient and tumor characteristics.

*No.*	*Sex*	*Age (years)*	*Tumor Location*	*GTV (cc)*
Overlap Group (n=11)				
1	Male	46	Left lobe	255.1
2	Female	58	Right posterior lobe	177.8
3	Male	51	Right posterior lobe	107.5
4	Male	58	Right anterior lobe	26.9
5	Male	41	Caudate lobe	70.7
6	Male	53	Caudate lobe	45.6
7	Male	47	Left medial lobe	86.9
8	Male	65	Left medial lobe	25.8
9	Female	56	Caudate lobe	36.4
10	Male	68	Left lobe	55.9
11	Male	50	Right posterior lobe	112.0
Nonoverlap Group (n=13)				
1	Female	59	Right posterior lobe	94.8
2	Male	54	Right posterior lobe	98.0
3	Male	53	Right anterior lobe	35.8
4	Female	45	Right anterior lobe	29.7
5	Male	45	Right posterior lobe	134.9
6	Female	65	Right anterior lobe	51.2
7	Male	37	Right anterior lobe	30.0
8	Male	44	Right anterior lobe	122.5
9	Male	69	Right posterior lobe	60.1
10	Female	57	Right anterior lobe	43.0
11	Male	54	Right posterior lobe	54.7
12	Male	67	Right lobe	72.5
13	Male	56	Right posterior lobe	24.2

GTV=gross tumor volume.

### B. Imaging, contouring, and planning objectives

During CT scanning, all patients were immobilized using vacuum bags in the supine position with their arms elevated above their head. Contrast‐enhanced 4D CT scans were acquired during uncoached quiet breathing at a 2.5 mm slice thickness on a 16‐slice positron emission tomography/CT system (GE Medical Systems, Waukesha, WI) as described previously.^8^, ^12^

Gross tumor volumes (GTVs) and clinical target volumes (CTVs) were manually contoured by a single clinician on all ten phases of the 4D CT scan using standard window/level settings. GTV represented the primary tumor visualized on the CT images; CTV was defined as the GTV plus an isotropic margin of 1.0 cm confined to the liver. Internal target volume (ITV) was defined as the sum of CTVs from the multiple 4D CT phases. An isotropic margin of 0.6 cm was added to the ITV to generate the PTV, to account for interfractional motion variability and daily setup errors. OARs included the liver, kidney, stomach, small intestine, and spinal cord. Normal liver volume was defined as the total liver volume minus the GTV. All contours were automatically projected onto the 20% CT dataset (mid‐exhalation) for dose calculation.

The prescribed dose for all plans was 50 Gy in daily fractions of 2.0 Gy with an inhomogeneity tissue correction. To avoid unnecessary biases in the optimization and evaluation processes, normalization was set to the PTV mean dose. The planning objectives for the PTV aimed to limit the minimal and maximal doses to 90% and 110% of the prescribed dose. The dose‐volume planning objectives for the OARs were defined as follows: normal liver, mean dose $<28\;Gy,V_{30Gy}<50\%$; stomach, maximal dose $<52\;Gy,V_{40Gy}<30\%$; small intestine, maximal dose <52Gy; bilateral kidney, mean dose $<18\;Gy,V_{20Gy}<30\%$; and spinal cord, maximal dose <45 Gy.

### C. Planning techniques

Two treatment plans were calculated for each patient, for use on an Elekta Synergy accelerator (MLCi2 with 80 leaves of 1 cm width; Elekta, Stockholm, Sweden) with 8 MV photons. The isocenter was positioned at the geometric center of the PTV. Both the 3D CRT and the VMAT plans were generated by the same professional medical dosimetrist to minimize interobserver variations.

#### C.1 3D CRT

3D CRT plans were generated utilizing the Pinnacle treatment planning system (TPS; Philips ADAC Pinnacle^3^ 8.0m, Philips Healthcare, Andover, MA) with a collapsed cone convolution (CCC) algorithm.^(13)^ Three to five coplanar beam arrangements with different wedge angles were manually optimized, and a fixed dose rate of 400 MU/min was used.

#### C.2 VMAT

VMAT planning was performed using the Monaco TPS (CMS version 3.0; Elekta, Crawley, UK), which was released clinically in 2010. Monaco uses a two‐stage process for optimizing dose distributions, offering equivalent uniform dose‐based biological optimization combined with physical and radiobiological cost functions, as previously described.^(8)^ The first stage was performed with the Pencil Beam dose calculation algorithm to obtain the ideal modulated fluence. In the second stage, the segments were optimized directly according to the machine parameters using the Monte Carlo (MC) algorithm for the final calculation.^(14)^ VMAT plans comprised a single 360° arc, allowing dose delivery with simultaneously varying gantry speed, multileaf collimator (MLC) leaf positions, and dose rate to optimize the dose distribution. Maximal leaf speed for the Elekta linear accelerator (linac) was 2.4 cm/sec and the maximal gantry speed was 6.0°/sec. There were seven available dose rates: 700, 350, 175, 88, 44, 22, and 11 MU/min. Test plans with different optimization objectives were generated, and then an experienced and senior radiation oncologist evaluated all VMAT plans to identify the optimal plan according to the planning objectives described below.

### D. Evaluation of treatment plans

Quantitative evaluation of the plans was performed using dose‐volume histograms (DVHs). For the PTV, $D_{1\%}$ (dose received by ≤1% of the volume) and $D_{99\%}$ values were defined as metrics for the maximal and minimal doses. $V_{95\%}$ (the volume receiving ≥95% of the prescribed dose), $V_{98\%}$, and $V_{107\%}$ were also reported. The conformity index (${CI}_{95\%}$) was defined as the ratio of the volume receiving ≥95% of the prescribed dose for the PTV. ${CI}_{80\%}$ and ${CI}_{60\%}$ were also reported to evaluate the dose gradient. The homogeneity index was expressed in terms of $D_{5\%}-D_{95\%}$, as defined by Bignardi et al.^(3)^ For OARs, the maximal dose, the mean dose, and a set of $V_{\text{xGy}}$ (volume receiving at least x Gy) values were scored. For the stomach and small intestine, the maximal dose was expressed as $D_{1cc}$.

Average cumulative DVHs for the PTV and OARs were built from the individual DVHs averaging the corresponding volumes over all cases for each dose bin of 0.01 Gy. Delivery time and MU/fraction were recorded to assess the efficiency of treatment delivery. The effective treatment time was measured at the linac, defined as the time for which the pure beam was on plus the time needed to reset the system between beams.

### E. Treatment accuracy

To evaluate the quality of VMAT delivery and the agreement between the dose calculations and treatment, the VMAT plans were verified dosimetrically using a Delta^4^ phantom (ScandiDos, Uppsala, Sweden), as described by Bedford et al.^(15)^ A correction factor was applied to the measurements to eliminate the effect of daily variations in output from the machine. The gamma evaluation criterion was ± 3% of 2 Gy and the distance to agreement was 3 mm, as commonly used in the clinic. Detectors measuring less than 0.2 Gy were excluded from the evaluation.

### F. Statistical analysis

The data were analyzed using the paired t‐test with SPSS 13.0 software. Differences were considered to be significant when the two‐tailed p‐value was less than 0.05.

## III. RESULTS

**Figure 1:**
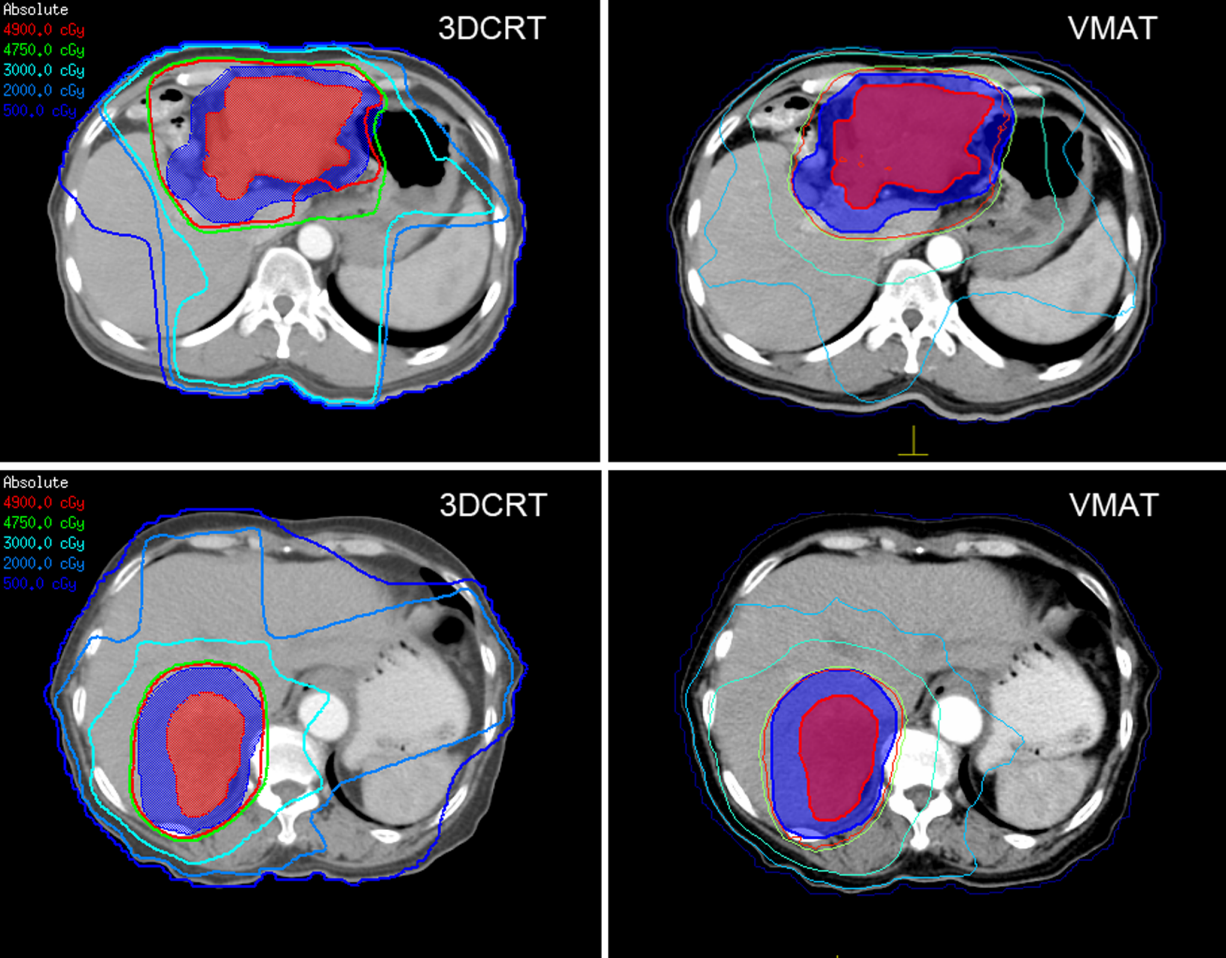
Typical dose distribution in axial view for two patients in each group. Red shading = GTV; blue shading = PTV.

The dose distributions for two typical patients are shown in the axial view in Fig. 1. 2, 3 display the average DVHs for the PTVs and OARs in each group. 2, 3 list the numeric findings from the DVH analysis of the PTVs and OARs, respectively.

**Figure 2:**
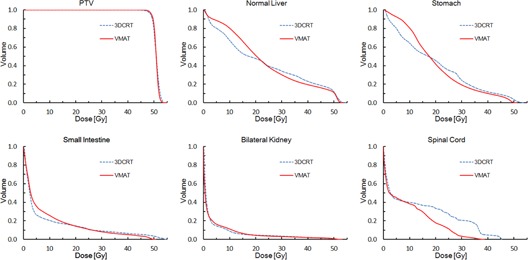
Average dose‐volume histograms of PTV and organs at risk for the adjacent group.

**Figure 3:**
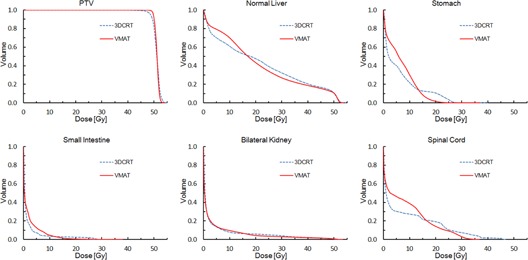
Average dose‐volume histograms of PTV and organs at risk for the nonadjacent group.

**Table 2:** Summary of averaged dosimetric results for PTV (n=24).

*Item*	*3D CRT*	*VMAT*	*p*
Mean (Gy)	50.0±0.0	50.0±0.0	
$D_{1\%}\;(Gy)$	53.0±0.7	53.0±0.3	0.907
$D_{99\%}\;(Gy)$	46.7±1.4	48.5±0.8	<0.001
$V_{95\%}\;(\%)$	98.2±1.2	99.6±0.4	<0.001
$V_{95\%}\;(\%)$	93.2±2.6	97.5±2.4	<0.001
$V_{107\%}\;(\%)$	1.2±2.2	0.3±0.3	0.082
HI (Gy)	3.8±1.0	2.8±0.5	0.001
${CI}_{95\%}$	1.4±0.1	1.3±0.1	0.003
${CI}_{80\%}$	2.1±0.4	1.8±0.1	0.003
${CI}_{60\%}$	4.0±1.1	2.8±0.2	<0.001

HI=homogeneity index; CI=conformity index; $D_{x\%}=\text{dose received by x}\%\text{of volume}$; $V_{x\%}=\text{volume receiving at least x}\%\text{of prescribed dose}$.

**Table 3:** Summary of averaged dosimetric results for organs at risk.

	*Adjacent Group* (n=11)			*Nonadjacent Group* (n=13)		
*Item*	*3D CRT*	*VMAT*	*p*	*3D CRT*	*VMAT*	*p*
Normal Liver: 1181.0±222.5 cc						
Mean (Gy)	22.0±2.9	23.3±2.0	0.112	21.3±4.7	21.9±3.9	0.514
$V_{30Gy}\;(\%)$	34.2±6.4	30.6±4.3	0.014	32.3±9.1	26.6±6.4	0.003
$V_{20Gy}\;(\%)$	46.5±11.7	51.3±8.5	0.159	47.1±13.6	43.2±9.2	0.177
$V_{10Gy}\;(\%)$	67.4±9.5	80.6±9.4	0.006	61.8±14.5	70.3±13.7	0.002
$V_{5Gy}\;(\%)$	80.5±10.0	88.0±9.3	0.004	70.7±14.3	79.6±13.4	0.002
Bilateral Kidney: 327.8±92.1 cc						
Mean (Gy)	3.4±2.7	3.3±2.8	0.448	2.8±3.3	2.8±2.7	0.993
$V_{20Gy}\;(\%)$	4.7±5.4	4.3±5.4	0.235	5.7±8.6	4.6±5.9	0.405
Stomach: 366.5±251.3 cc						
$D_{1cc}\;(Gy)$	45.9±8.7	42.9±9.9	<0.001	18.6±8.1	19.8±5.6	0.619
Mean (Gy)	19.9±8.3	19.9±8.7	0.973	6.3±4.0	6.7±2.2	0.717
$V_{40Gy}\;(\%)$	11.5±12.1	10.3±10.6	0.108	0	0	
$V_{30Gy}\;(\%)$	24.6±19.4	20.0±18.4	0.090	0.0±0.0	0.0±0.1	0.082
Small Intestine: 263.0±190.3 cc						
$D_{1cc}\;(Gy)$	47.0±7.2	45.2±6.4	0.002	12.6±10.6	13.2±11.1	0.607
$V_{45Gy}\;(cc)$	8.4±10.9	5.9±6.5	0.173	0	0	
$V_{15Gy}\;(cc)$	38.4±33.0	41.4±29.0	0.491	4.9±14.1	6.2±4.3	0.653
Spinal Cord: 22.9±5.7 cc						
Max (Gy)	33.3±8.0	24.9±7.4	0.014	28.5±9.9	23.0±7.5	0.131

### A. Target coverage and dose homogeneity

As shown in Table 2, in the whole group compared with the 3D CRT plans, the VMAT plans provided a statistically significant improvement in PTV dose coverage, conformity, and homogeneity. There was slightly better sparing of the hot spot of the PTV ($V_{107\%}$) with VMAT than with 3D CRT (0.3% vs. 1.2%, p=0.082), but statistical significance was not reached. Additionally, VMAT had a steeper dose gradient than 3D CRT in each group (2, 3).

### B. OARs

#### B.1 Adjacent group

All VMAT plans were shown to be capable of achieving the planning objectives. In contrast, the 3D CRT plans exceeded the dose constraints for the stomach and/or small intestine in five patients. As shown in Table 3, compared with 3D CRT, VMAT provided superior sparing of all serial functioning normal tissues, including the stomach, small intestine, and spinal cord. The maximal dose to the stomach and small intestine was decreased by 3.0±1.8 (p<0.001) and 1.8±1.4 Gy (p=0.002), respectively, in the VMAT plans.

Although the high‐dose region of normal liver ($V_{30Gy}$) was lower with VMAT than with 3D CRT, the low‐dose regions ($V_{10Gy}$ and $V_{5Gy}$) with VMAT were significantly higher than that with 3D CRT. The mean dose to normal liver (MDTNL) was slightly higher with VMAT plans than with 3D CRT plans, but with no statistical significance.

#### B.2 Nonadjacent group

Both VMAT and 3D CRT plans achieved the planning objectives. Although VMAT resulted in a lower dose to the spinal cord, sparing of most OARs, including the liver, stomach, and small intestine (Table 3) was inferior, though statistical significance was not reached. Similar to the dose distribution in the adjacent group, the low‐dose regions ($V_{10Gy}$ and $V_{5Gy}$) were significantly higher with VMAT than with 3D CRT. No significant difference in MDTNL was observed between the two plans (p=0.514).

### C. Delivery parameters and treatment accuracy

The MU/fraction for VMAT (434±67) was slightly higher than that for 3D CRT (391±92), with an average increase of 11.3% (p=0.062) in the whole group. The effective treatment times were as follows: 2.1±0.3 min for 3D CRT and 3.1±0.2 min for VMAT (p<0.001). Comparison of planned and measured cross‐sectional dose planes indicated high conformity for VMAT delivery, with an average gamma evaluation passing rate of 98.6%±0.4%.

## IV. DISCUSSION

Although several dosimetric comparisons of IMRT/VMAT with 3D CRT for liver lesions have been published, it remains unclear which technique is the optimal strategy for HCC.^9^, ^10^, ^11^ Lee et al.^(16)^ compared 3D CRT, IMRT, and helical tomotherapy for HCC according to tumor location (left lobe, right lobe, or both lobes); however, they could not reach any definitive conclusions. We assume that comparisons based on the anatomical location of tumor in liver lobes or segments are inappropriate. To clarify this issue, in the present study we compared VMAT with 3D CRT according to the relationship between liver lesions and the alimentary tract.

VMAT provided improved target coverage, conformity, and homogeneity compared with conformal irradiation in both the adjacent group and the nonadjacent group. However, the dosimetric advantage of VMAT over 3D CRT was not identical across OARs in the two groups. In the adjacent group, the major benefit of VMAT was the reduction of dose to the stomach, small intestine, and spinal cord. In the nonadjacent group, clinical gains were seen only in the dose to the spinal cord.

In patients with abdominal lesions overlapping or adjacent to the alimentary tract, radiation‐induced gastrointestinal complications due to excess radiation are severe. Kavanagh et al.^(17)^ reported that a dose of the order of 50 Gy was associated with 2%–6% and 2%–9% risks of severe late radiation‐induced toxicity to the stomach and small bowel, respectively. According to published data, the maximal dose and the volume of alimentary tract receiving higher doses should be minimized in the plan. In the adjacent group in the present study, VMAT plans showed superior sparing of the stomach and small intestine compared with 3D CRT, in terms of maximal dose, mean dose, and the volume receiving a high dose (>30 Gy). Dose reduction to the alimentary tract could be associated with decreased clinical toxicity, which has been demonstrated by Yovino et al.^(6)^ Further research is needed to evaluate the clinical impact of the improved dose–volumetric results achieved with VMAT in our study. Additionally, our results indicate that VMAT offers potential for dose escalation with stable normal tissue complication probabilities, and this possibility demands further investigation.

Radiation‐induced liver disease (RILD) is a serious complication of hepatic irradiation. With improvements in conformal radiotherapy, our understanding of the relationship between liver dose and volume and the risk of RILD has improved considerably. Previous studies have indicated that MDTNL is a strong predictor of subsequent RILD.^1^, ^18^ Data from the University of Michigan Medical Center showed that the MDTNL associated with a 5% risk of classic RILD was 28 Gy for primary and 32 Gy for metastatic liver cancer.^(19)^ Kim et al.^(20)^ also demonstrated a significant correlation of MDTNL with RILD. In contrast, effectiveness of $V_{5Gy}-V_{30Gy}$ in predicting the risk of RILD is not uniformly observed.^1^, ^18^ In the present study, the normal liver was the main dose‐limiting structure in the nonadjacent group and VMAT failed to decrease the MDTNL compared with 3D CRT, a finding consistent with previous reports.^9^, ^16^ Theoretically, based on the relationship between MDTNL and RILD, the benefit of VMAT over 3D CRT in the nonadjacent group is limited and dose escalation in VMAT plans is rather difficult. Therefore, the evidence that 3D CRT should be replaced by VMAT when treating liver lesions away from the alimentary tract is not convincing; in these cases, 3D CRT and VMAT should be evaluated on a case‐by‐case basis.

It has been documented that the major advantages of VMAT over IMRT are the lower number of monitor units and the higher delivery efficiency, with a reduction in treatment time of 35%–61%.^3^, ^4^, ^5^, ^21^ Otto^(22)^ reported that a 2 Gy fraction can generally be delivered in 1.5–3 min with VMAT. In our study, the average treatment time was 3.1±0.2 min for VMAT, which is slightly longer than that reported for VMAT delivered using RapidArc (Varian Medical Systems, Palo Alto, CA) on a Varian linac.^(4)^ This difference is mainly related to the binned dose rate mode on Elekta accelerators, which have only seven fixed dose rates instead of the continuous dose rate shifts provided by the Varian linac.

One concern with the use of VMAT is the dosimetric effects of respiratory motion in patients with thoracic or abdominal malignancies. Application of VMAT to liver tumors needs to address the interplay between the moving organ and the dynamic treatment device. Kuo et al.^(23)^ reported that, in patients with respiratory motion of less than 1.5 cm, the dosimetric impact is rather small in VMAT, for both single and multiple fractionations. Rao et al.^(24)^ also suggested that 4D CT‐based VMAT plans experience negligible interplay effects between the MLC sequence and tumor motion. Based on the published data, we selected HCC patients with low and medium breathing motion amplitude (<1.5 cm) in the craniocaudal direction, as assessed on 4D CT images.

Because the alimentary tract can move unpredictably regardless of respiration, there is a concern that motion of hollow viscera during treatment may introduce uncertainty into the dose distribution. However, we used the same 4D CT dataset for both techniques, and therefore the dosimetric comparison between 3D CRT and VMAT plans was not affected by organ motion.

A potential limitation of our study is the use of two different dose calculation algorithms performed by two different TPSs for comparative analysis. The reason is that VMAT plans generated by the Pinnacle TPS (Philips ADAC Pinnacle3 8.0m) are currently unavailable at our institute and the Monaco TPS (version 3.0) is not capable of generating 3D CRT plans. In addition, the two planning systems use different dose calculation algorithms. However, Calvo et al.^(25)^ reported that the mean dose difference between the CCC and MC algorithms for 88 lung plans was 1.38%. Fotina et al.^(26)^ also demonstrated only small deviations in PTV dose (1%–2%) between the MC and CCC algorithms in IMRT. In accordance with the standard protocol at our institution, each TPS was verified quantitatively for a small field size by both ionization chamber and Kodak extended dose range film (EDR2) measurements before its clinical implementation. Our own work confirmed that the dose distributions obtained from the CCC almost agreed with those from the MC (deviations less than 1.6% for the abdominal region), a finding consistent with previous reports.^25^, ^26^, ^27^

## V. CONCLUSIONS

Compared with 3D CRT, VMAT provided improved target coverage, conformity, and homogeneity over the whole group of patients with HCC. In patients with liver lesions overlapping or adjacent to the alimentary tract, VMAT plans should be preferred on account of the plan quality, delivery efficiency, and superior sparing of stomach and small intestine. However, for liver lesions distal to the alimentary tract (i.e., <1.5 cm), VMAT plans did not provide any distinct advantage in terms of normal tissue protection. In such cases, 3D CRT and VMAT should be evaluated on an individual basis.

## ACKNOWLEDGMENTS

This work was supported by grants from the Sci‐Tech Project Foundation of Guangdong Province (No. 2012B031800287).
